# β-Thujaplicin Enhances TRAIL-Induced Apoptosis via the Dual Effects of XIAP Inhibition and Degradation in NCI-H460 Human Lung Cancer Cells

**DOI:** 10.3390/medicines8060026

**Published:** 2021-06-02

**Authors:** Saki Seno, Minori Kimura, Yuki Yashiro, Ryutaro Kimura, Kanae Adachi, Aoi Terabayashi, Mio Takahashi, Takahiro Oyama, Hideaki Abe, Takehiko Abe, Sei-ichi Tanuma, Ryoko Takasawa

**Affiliations:** 1 Faculty of Pharmaceutical Sciences, Tokyo University of Science, 2641 Yamazaki, Noda, Chiba 278-8510, Japan; saki_ss17@yahoo.co.jp (S.S.); 3a17042@ed.tus.ac.jp (M.K.); yuki-ysr.0527@outlook.jp (Y.Y.); ryutaro.k.pharma@gmail.com (R.K.); a-k18@outlook.com (K.A.); 3a17083@ed.tus.ac.jp (A.T.); 3a17072@ed.tus.ac.jp (M.T.); 2 Hinoki Shinyaku Co. Ltd., Chiyoda-ku, Tokyo 102-0084, Japan; takahiro.oyama@hinoki.co.jp (T.O.); hideaki.abe@hinoki.co.jp (H.A.); mugino.abe@hinoki.co.jp (T.A.); 3 Department of Genomic Medicinal Science, Research Institute for Science and Technology, Organization for Research Advancement, Tokyo University of Science, 2641 Yamazaki, Noda, Chiba 278-8510, Japan; tanuma@rs.tus.ac.jp

**Keywords:** β-thujaplicin, XIAP, TRAIL, apoptosis, cancer, NCI-H460 cells

## Abstract

**Background**: β-thujaplicin, a natural tropolone derivative, has anticancer effects on various cancer cells via apoptosis. However, the apoptosis regulatory proteins involved in this process have yet to be revealed. **Methods:** Trypan blue staining, a WST-8 assay, and a caspase-3/7 activity assay were used to investigate whether β-thujaplicin sensitizes cancer cells to TNF-related apoptosis-inducing ligand (TRAIL)-mediated apoptosis. Additionally, western blotting was performed to clarify the effects of β-thujaplicin on X-linked inhibitor of apoptosis protein (XIAP) in NCI-H460 cells and a fluorescence polarization binding assay was used to evaluate the binding-inhibitory activity of β-thujaplicin against XIAP-BIR3. **Results:** β- and γ-thujaplicins decreased the viability of NCI-H460 cells in a dose-dependent manner; they also sensitized the cells to TRAIL-induced cell growth inhibition and apoptosis. β-thujaplicin significantly potentiated the apoptosis induction effect of TRAIL on NCI-H460 cells, which was accompanied by enhanced caspase-3/7 activity. Interestingly, β-thujaplicin treatment in NCI-H460 cells decreased XIAP levels. Furthermore, β-thujaplicin was able to bind XIAP-BIR3 at the Smac binding site. **Conclusions:** These findings indicate that β-thujaplicin could enhance TRAIL-induced apoptosis in NCI-H460 cells via XIAP inhibition and degradation. Thus, the tropolone scaffold may be useful for designing novel nonpeptidic small-molecule inhibitors of XIAP and developing new types of anticancer drugs.

## 1. Introduction

β-thujaplicin, also known as hinokitiol, is a natural tropolone derivative with a β-diketone structure in its molecule; it forms metal chelates in the presence of various metal ions including ferric ion (Fe^2+^), copper ion (Cu^2+^), and zinc ion (Zn^2+^) [1]. β-thujaplicin is used in various personal care products (e.g., cosmetics, body soaps, and toothpastes) and exhibits a variety of biological properties including antibacterial [2,3], antifungal [4], antiviral [5], and anticancer activities [6,7,8,9,10,11,12,13]. It is also reported to induce apoptosis in human cancer cells, such as human colon cancer cells [9] and hepatocellular carcinoma cells [13]; however, the mechanism and targets of β-thujaplicin’s anticancer activity have yet to be fully elucidated.

X-linked inhibitor of apoptosis protein (XIAP), the most potent member of the inhibitor of apoptosis protein (IAP) family of endogenous caspase inhibitors, blocks the initiation and execution phases of the apoptotic cascade through inhibition of caspases-9, -3, and -7 [14]. XIAP contains three baculoviral IAP repeat (BIR) motifs; each BIR domain folds into a functionally independent structure that binds a zinc ion. Additionally, XIAP contains another zinc-binding motif, the RING domain, which has E3 ubiquitin ligase activity [14]. Of all the IAP family members, XIAP is the only one able to directly inhibit both the initiation and execution phases of the caspase cascade [14]. The BIR2 domain of XIAP binds and inhibits caspases-3 and -7, whereas the BIR3 domain binds and inhibits caspase-9 (which is an apical caspase in the mitochondrial pathway of apoptosis). XIAP levels are elevated in many cancer cell lines and suppression of XIAP protein expression sensitizes cancer cells to chemotherapeutic agents [15,16,17]. Thus, XIAP represents an attractive target for the development of apoptosis-resistant cancer treatments. The inhibition or decrease of XIAP in cancer cells lowers the apoptotic threshold, which in turn induces cell death and/or enhances the cytotoxic action of chemotherapeutic agents.

It was previously reported that treatment with the membrane-permeable zinc chelator N,N,N’,N’,-tetrakis(2-pyridylmethyl) ethylenediamine (TPEN) induced depletion of XIAP at the posttranslational level in human PC-3 prostate cancer cells while sensitizing these cells to TNF-related apoptosis-inducing ligand (TRAIL)-mediated apoptosis [18]. These findings indicate that zinc-chelating agents could be used to sensitize malignant cells to apoptosis-inducing agents via reduction of XIAP. Since β-thujaplicin acts as a chelator of zinc ions [1], it may play an important role in the regulation of XIAP-mediated apoptosis. However, the effect of β-thujaplicin on XIAP has yet to be examined.

The BIR3 domain of XIAP, where caspase-9 and Smac proteins bind, is considered an attractive site for the design of small-molecule XIAP inhibitors. Embelin, a natural compound from the Japanese Ardisia herb, is one of the cell-permeable, nonpeptide, small-molecular weight inhibitors that target the XIAP-BIR3 domain as identified through computational structural screening of the herbal medicine compounds database [19]. It binds to the XIAP-BIR3 domain at the position where Smac and caspase-9 also bind and thereby antagonize XIAP-BIR3 (IC_50_ value = 4.1 µM) [19]. Embelin consists of a di-hydroxyquinone core and long hydrophobic tail, whereas β-thujaplicin has a seven-membered carbon ring and an isopropyl side chain; however, both compounds have similar aromatic rings with α-hydroxy ketone and an alkyl group.

Based on the findings detailed above, we hypothesize that β-thujaplicin could act on XIAP both as a zinc chelator and as an inhibitor targeting the XIAP-BIR3 domain to induce and/or enhance apoptosis. To test this hypothesis, we examined the apoptosis inducibility of β-thujaplicin in combination with TRAIL in human non-small cell lung cancer NCI-H460 cells. NCI-H460 cells are considered type II cells, which are dependent on the mitochondrial apoptotic pathway to amplify effector caspase activation during death receptor-induced apoptosis [20]. XIAP is highly expressed in NCI-H460 cells, which show chemoresistance, and binds to the processed form of caspase-9 before suppressing the activation of downstream effector caspases-3 and -7 [21]. Therefore, we investigated whether β- and γ-thujaplicins could sensitize NCI-H460 cells to TRAIL-induced apoptosis. This research is the first to demonstrate that β-thujaplicin enhances TRAIL-induced apoptosis in NCI-H460 cells. Furthermore, we reveal that β-thujaplicin can bind the XIAP-BIR3 domain at the Smac binding site. Moreover, we show that β-thujaplicin treatment decreases the amount of XIAP protein in NCI-H460 cells. Our results validate β-thujaplicin as a potent seed compound that exerts dual effects on XIAP, i.e., XIAP inhibition by binding to the BIR3 domain and XIAP reduction by degrading XIAP protein. Thus, β-thujaplicin represents a new scaffold for the development of novel types of XIAP-targeting anticancer drugs.

## 2. Materials and Methods

### 2.1. Materials

Tropolone and β-thujaplicin were purchased from Tokyo Chemical Industry Co., Ltd. (Tokyo, Japan). γ-thujaplicin was purchased from Osaka Organic Chemical Industry Ltd. (Osaka, Japan). They were of analytical grade (purity >95%). An antibody against XIAP as well as anti-rabbit and anti-mouse immunoglobulin G (IgG) horseradish peroxidase (HRP)-coupled secondary antibodies were purchased from Cell Signaling Technology (Boston, MA, USA). An antibody against β-actin was purchased from FUJIFILM Wako Pure Chemical Corporation (Osaka, Japan).

### 2.2. Cell Culture

Cells of the human non-small cell lung cancer cell line NCI-H460 were maintained in RPMI1640 supplemented with 100 U/mL of penicillin, 100 µg/mL of streptomycin, and 10% fetal bovine serum. The cells were grown at 37 °C in a humidified atmosphere containing 5% CO_2_.

### 2.3. Trypan Blue Staining

The effects of tropolone derivatives on cell viability were evaluated using trypan blue staining. NCI-H460 cells were seeded in dishes and cultured overnight. The cells were then treated with tropolone derivatives alone or cotreated with SuperKillerTRAIL™, Soluble (human) (rec.) (AdipoGen Life Sciences, San Diego, CA, USA) for 24 h. After treatment, the cells were trypsinised and resuspended in complete medium. Each sample was then mixed with trypan blue solution. Colored (dead) and dye-excluding (viable) cells were counted using a Countess II Cell Counter (Thermo Fisher Scientific K.K., Tokyo, Japan).

### 2.4. Measurements of Cell Viability

Enhancement of the antiproliferative effect of TRAIL on NCI-H460 cells through a combination treatment with β- or γ-thujaplicin was evaluated by measuring cell growth inhibition. The number of viable cells was estimated via a WST-8 assay using a Cell Counting Kit-8 according to the manufacturer’s instructions (Dojindo Laboratories, Kumamoto, Japan).

### 2.5. Evaluation of the Combinatorial Effects of Drugs

The combinatorial effects of TRAIL and β- or γ-thujaplicin were evaluated using an excess-over-Bliss additivism (EOBA) model [22,23] with the following Formula:

EOBA = C − [A + B − (A × B)],

where C is equal to the fractional inhibition of both drugs simultaneously, A is equal to the fractional inhibition of drug A, and B is equal to the fractional inhibition of drug B, while fractional inhibition is equal to 1.0 minus the viability (expressed as a value from 0.0 to 1.0). Positive, negative, and near-zero EOBA values reflect synergistic, antagonistic, and additive drug combinations, respectively.

### 2.6. Caspase-3/7 Activity Assay

To analyze caspase activity, NCI-H460 cells were seeded in a 96-well luminometer plate and cultured overnight. The cells were treated with TRAIL or β-thujaplicin alone and cotreated with both reagents for 24 h. A Caspase-Glo^®^ 3/7 Assay was performed according to manufacturer’s protocol (Promega, Madison, WI, USA).

### 2.7. Western Blotting

Cell lysates were prepared using Cell Lysis Buffer (Cell Signaling Technology, Boston, MA, USA). Samples containing equal amounts of protein were separated by SDS-PAGE and transferred to nitrocellulose membranes. The membranes were blocked with 5% skim milk and 0.25% bovine serum albumin in Tris-buffered saline containing 0.1% Tween 20 (TTBS) for 1 h at room temperature and then probed with appropriate primary antibodies overnight at 4 °C. Subsequently, washed with TTBS, and then incubated with the appropriate secondary antibody for 1 h at room temperature. After washing, the blotted proteins were visualized with an iBright Western Blot Imaging System (Thermo Fisher Scientific K.K., Tokyo, Japan) using ImmunoStar LD (FUJIFILM Wako Pure Chemical Corporation, Osaka, Japan).

### 2.8. Expression and Purification of Recombinant GST-Tagged XIAP-BIR3 Domain in an Escherichia coli Expression System

A human XIAP-BIR3 cDNA fragment was subcloned into pGEX-4T-2 (GE Healthcare, Chicago, IL, USA), and the resulting construct was transformed into BL21 to produce recombinant human XIAP-BIR3 as a GST-tagged protein. The recombinant GST-tagged XIAP-BIR3 domain was purified with Glutathione Sepharose^TM^ 4B (GE Healthcare, Chicago, IL, USA) in native conditions following the manufacturer’s protocol.

### 2.9. Fluorescence Polarization Binding Assay

The interaction between the Smac peptide and the BIR3 domain of XIAP was measured using a fluorescence polarization assay, which was performed at room temperature in 384-well black polystyrene microplates (Thermo Fisher Scientific K.K., Tokyo, Japan) on an EnVision^®^ 2104 multimode plate reader (PerkinElmer, Buckinghamshire, UK). PBS (−) supplemented with 0.05% Tween 20, 1 nM of 5-FAM labeled Smac/Diablo Peptide [AVPIAQKSE] (AnaSpec, Fremont, CA, USA) as a fluorescent probe, and 50 nM of recombinant GST-tagged XIAP-BIR3 domain were used for all measurements.

## 3. Results

### 3.1. β-. and γ-Thujaplicins Decrease the Viability of NCI-H460 Cells

**Figure 1:**
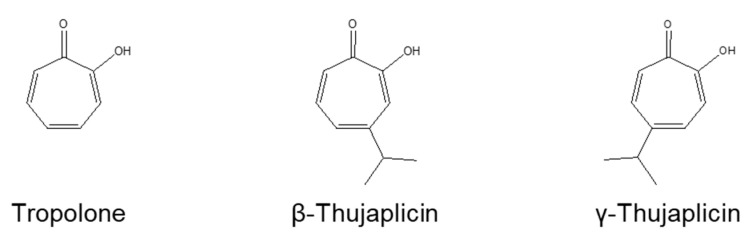
The structures of tropolone, β-thujaplicin, and γ-thujaplicin.

**Figure 2:**
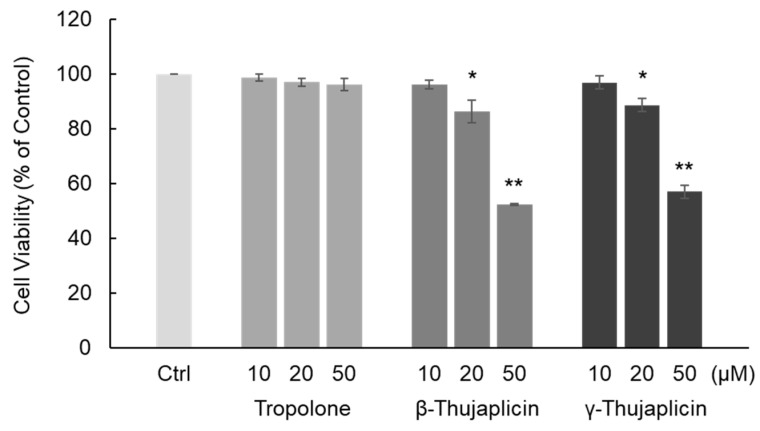
Quantitation of cell viability by trypan blue staining following tropolone, β-thujaplicin, and γ-thujaplicin treatments on NCI-H460 cells at the indicated concentrations for 24 h. Cell viability (% of control) was measured using a trypan blue exclusion assay. Data are the averages of three independent experiments and error bars show the standard deviation. * *p* < 0.05 and ** *p* < 0.01 compared with untreated NCI-H460 cells (Ctrl).

First, we examined the cytotoxic effect of tropolone, β-thujaplicin, and γ-thujaplicin on NCI-H460 cells using a trypan blue exclusion assay. The chemical structures of these compounds are shown in Figure 1. As shown in Figure 2, β- and γ-thujaplicin treatments for 24 h decreased the viability of NCI-H460 cells in a dose-dependent manner. Treatments with >20 μM β- and γ-thujaplicins significantly decreased the viability of NCI-H460 cells, whereas treatments at 10 μM did not affect cell viability. Tropolone did not affect NCI-H460 cells at doses up to 50 μM.

### 3.2. β-. and γ-Thujaplicins Enhance the Antiproliferative Effect of TRAIL on NCI-H460 Cells

**Figure 3:**
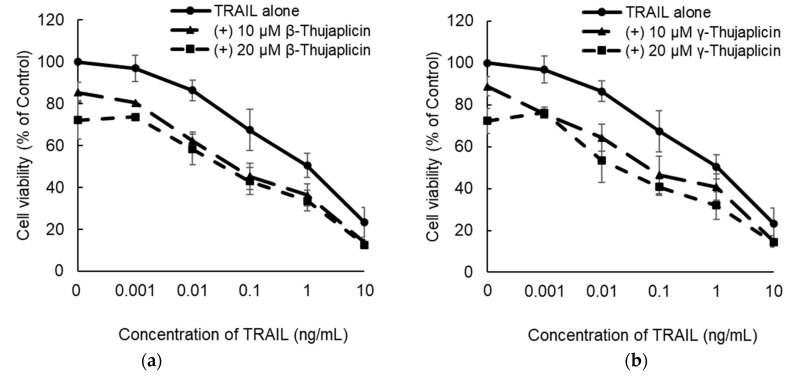

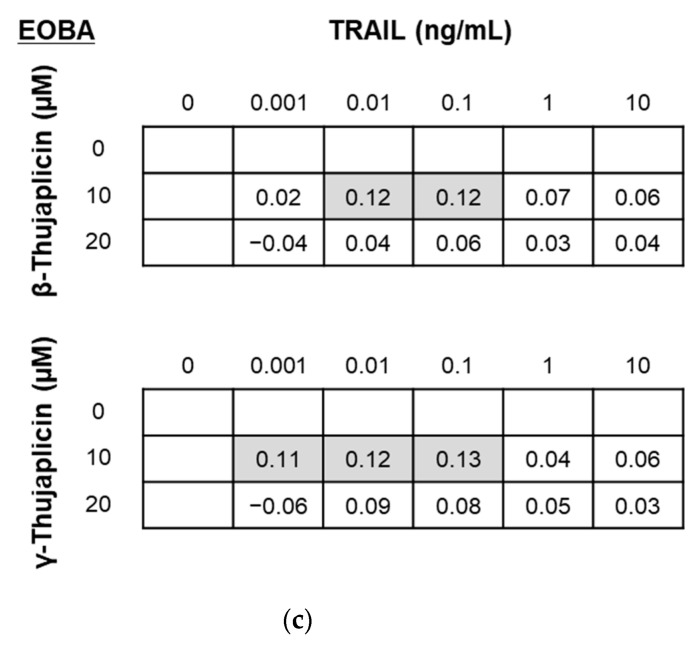
Enhancement of the antiproliferative effect of TRAIL on NCI-H460 cells by combination treatments with β- or γ-thujaplicin. NCI-H460 cells were cotreated with the indicated concentrations of TRAIL and β-thujaplicin (**a**) or γ-thujaplicin (**b**) for 24 h. Cell viability (% of control) was measured using a WST assay and EOBA scores were calculated (**c**). Data are the averages of three independent experiments and error bars show standard deviations.

NCI-H460 cells were cotreated with TRAIL and β-thujaplicin (Figure 3a) or γ-thujaplicin (Figure 3b) for 24 h; cell viability (% of control) was then measured using a WST assay and EOBA scores were calculated. As EOBA values >0.1 denote a synergistic combination [22,23], the data indicated that cotreatment with a relatively low concentration of TRAIL and 10 μM β- or γ-thujaplicin produced a synergistic antiproliferative effect on NCI-H460 cells (Figure 3c).

### 3.3. β-. Thujaplicin Enhances the Cell Death Induction Effect of TRAIL on NCI-H460 Cells

**Figure 4:**
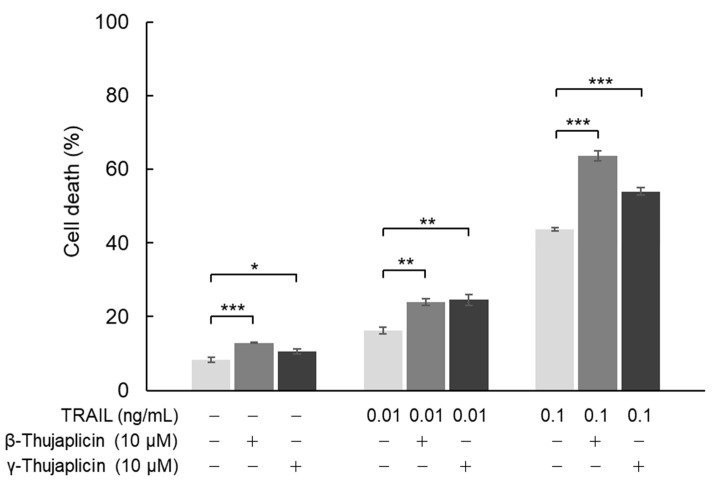
Enhancement of the TRAIL cell death induction effect on NCI-H460 cells through a combination treatment with β-thujaplicin. NCI-H460 cells were cotreated with the indicated concentrations of TRAIL and β- or γ-thujaplicin for 24 h. Cell death (%) was determined by a trypan blue exclusion assay. Data are the averages of three independent experiments and error bars show standard deviations. Asterisks (*, **, and ***) indicate *p* value smaller than 0.05 (*p* < 0.05), 0.01 (*p* < 0.01), and 0.001 (*p* < 0.001), respectively.

In NCI-H460 cells, we also investigated the cell death induction effect of combination treatments with β- or γ-thujaplicin and TRAIL using a trypan blue exclusion assay. As shown in Figure 4, 10 μM β-thujaplicin substantially potentiated cell death induction in NCI-H460 cells (to ~60%) in the presence of 0.1 ng/mL TRAIL. On the other hand, γ-thujaplicin only slightly enhanced TRAIL-induced cell death. Both thujaplicins had a small potentiating effect on cell death induced by 0.01 ng/mL TRAIL.

### 3.4. Potentiating Effect of β-Thujaplicin on TRAIL-Induced Cell Death in NCI-H460 Cells Is Accompanied by Enhanced Caspase-3/7 Activity

**Figure 5:**
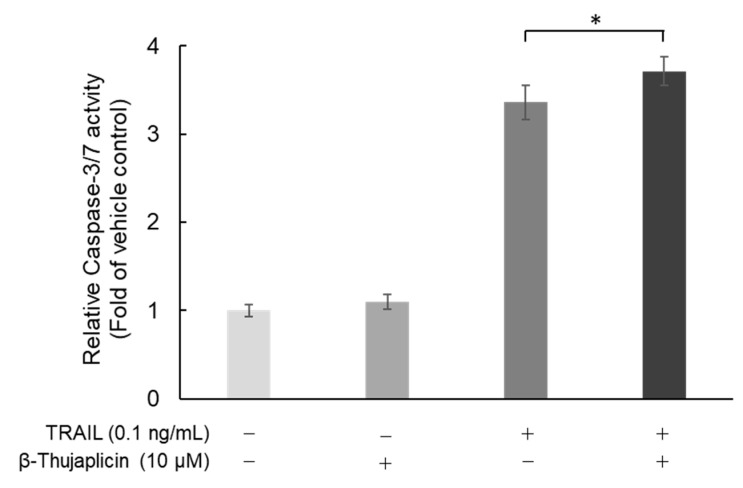
Enhancement of caspase-3/7 activity in TRAIL-treated NCI-H460 cells through combination treatment with β-thujaplicin. NCI-H460 cells were cotreated with 0.1 ng/mL TRAIL and 10 µM β-thujaplicin for 24 h. Caspase-3/7 activity was then measured by a Caspase-Glo^®^ 3/7 Assay System. Data are the averages of three independent experiments and error bars show standard deviations. * *p* < 0.05.

Next, we assessed whether cotreatment with β-thujaplicin promotes activation of caspase-3/7, which occurs during TRAIL-induced cell death in NCI-H460 cells. As shown in Figure 5, β-thujaplicin significantly enhanced caspase-3/7 activity in NCI-H460 cells in the presence of 0.1 ng/mL TRAIL.

### 3.5. Tropolone, β-Thujaplicin, and γ-Thujaplicin Treatments Decrease the Amount of XIAP Protein in NCI-H460 Cells

**Figure 6:**
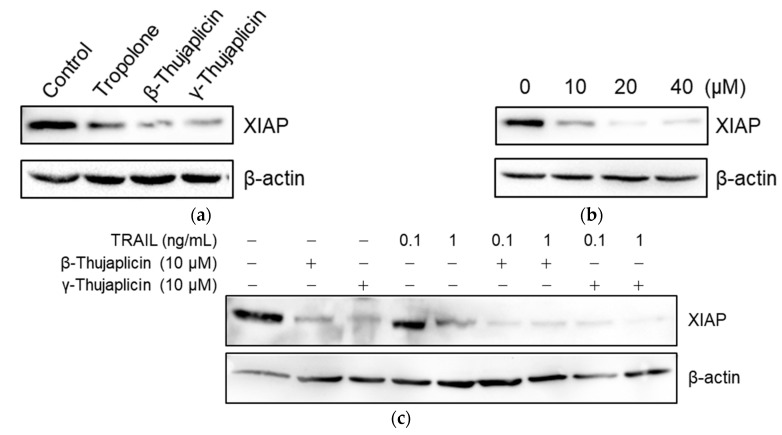
Decrease in XIAP protein levels in NCI-H460 cells following tropolone, β-thujaplicin, and γ-thujaplicin treatments. (**a**) NCI-H460 cells were treated with 10 µM of tropolone, β-thujaplicin, and γ-thujaplicin for 24 h. (**b**) NCI-H460 cells were treated with 0, 10, 20, and 40 µM of β-thujaplicin for 24 h. (**c**) NCI-H460 cells were cotreated with the indicated concentrations of TRAIL and β-thujaplicin or γ-thujaplicin for 24 h. In (**a**–**c**), cell lysates were subjected to western blot analysis using XIAP antibody.

To examine whether tropolone derivatives (which are zinc chelators) decrease XIAP protein levels in cells, NCI-H460 cells were treated with 10 µM of tropolone, β-thujaplicin, and γ-thujaplicin for 24 h, and then XIAP protein levels were measured in cell extracts via western blot analysis. Interestingly, the amount of XIAP protein in cells was reduced by 10 µM of tropolone derivatives, especially by β- and γ-thujaplicins (Figure 6a). Furthermore, the β-thujaplicin-mediated decrease in XIAP was dose-dependent (Figure 6b). As shown in Figure 6c, treatment with TRAIL alone at 0.1 ng/mL did not affect the amount of XIAP in cells; however, at 1 ng/mL, TRAIL treatment markedly decreased XIAP protein levels. When cotreated with 0.1 ng/mL TRAIL and 10 µM β- or γ-thujaplicin, the decrease in XIAP levels was considerably enhanced.

### 3.6. Tropolone, β-Thujaplicin, and γ-Thujaplicin Inhibit the Binding of the Smac N-Terminal Peptide to the XIAP-BIR3 Domain

Since the core structure of embelin is similar to that of tropolone, we evaluated the inhibitory effect of tropolone derivatives on the binding of the Smac N-terminal peptide to the XIAP-BIR3 domain using a fluorescence polarization assay. Under our experimental conditions, the IC_50_ value for embelin was 3.1 ± 0.6 µM (Table 1), similar to the previously reported IC_50_ value [19]. As shown in Table 1, tropolone, β-thujaplicin, and γ-thujaplicin inhibited the binding of the Smac N-terminal peptide to the XIAP-BIR3 domain in a dose-dependent manner. The IC_50_ values of tropolone, β-thujaplicin, and γ-thujaplicin were 83.1 ± 6.9 µM, 58.5 ± 2.8 µM, and 56.2 ± 2.9 µM, respectively. The inhibitory activities of β- and γ-thujaplicins were higher than that of tropolone.

## 4. Discussion

β-Thujaplicin is reported to exhibit anticancer activity in various human cancer cell lines [6,7,8,9,10,11,12,13]. In relation to this anticancer activity, β-thujaplicin has been shown to induce apoptosis in human colon cancer cells through the activation of caspases-9 and -3 [9] and in human hepatocellular carcinoma cells through a mitochondria-dependent intrinsic pathway [13]. Recently, it was reported that nontoxic doses of β-thujaplicin inhibit the migration of A549 lung cancer cells through suppression of matrix metalloproteinases and induction of apoptosis via caspases-9 and -3 activation [12]. To date, however, the mechanism and targets of β-thujaplicin’s anticancer activity have not been fully elucidated.

In the present study, we hypothesized that β-thujaplicin acts on XIAP both as a zinc chelator and an inhibitor targeting the XIAP-BIR3 domain to induce and/or enhance apoptosis. We used NCI-H460 cells to test this hypothesis because they are considered type II cells that depend on the mitochondrial apoptotic pathway to amplify effector caspase activation during death receptor-induced apoptosis [20]. We assumed that, if β-thujaplicin decreases and/or inhibits XIAP, it would enhance TRAIL-induced apoptosis via the liberation of caspases-9, -3, and -7 from XIAP-mediated inhibition.

We found that β- and γ-thujaplicins, but not tropolone, treatments decreased the viability of NCI-H460 cells in a dose-dependent manner. These results suggest that an alkyl side chain, in this case an isopropyl group, attached to tropolone contributes to the cell death induction abilities of the tropolone derivatives. We therefore investigated whether β- and γ-thujaplicins could sensitize NCI-H460 cells to TRAIL-induced apoptosis. We found that 10 µM of β- and γ-thujaplicins alone did not induce a significant degree of cytotoxicity and cell death, whereas a combined treatment of TRAIL and β- or γ-thujaplicin resulted in a synergistic antiproliferative effect on NCI-H460 cells. Furthermore, 10 µM of β-thujaplicin substantially potentiated the cell death induction effect of 0.1 ng/mL TRAIL on NCI-H460 cells. Thus, we further investigated β-thujaplicin as a cotreatment, particularly whether it could promote activation of caspase-3/7, which occurs during TRAIL-induced apoptosis in NCI-H460 cells. β-Thujaplicin cotreatment with TRAIL was shown to significantly enhance the increase in caspase-3/7 activity in NCI-H460 cells. To our knowledge, this is the first report of β-thujaplicin promoting TRAIL-induced apoptosis by enhancement of caspase-3/7 activity in cancer cells.

XIAP is probably the only bona fide inhibitor that selectively binds and inhibits caspases-9, -3, and -7, with zinc-binding BIR domains being responsible for this inhibitory function [14]. We found that a β-thujaplicin-mediated reduction in XIAP levels coincides with enhanced cell death and caspase-3/-7 activity in NCI-H460 cells treated with TRAIL. When NCI-H460 cells were treated with β-thujaplicin alone, the level of caspases-3/-7 activity remained consistently low throughout 24-h incubation. Although treatment of NCI-H460 cells with TRAIL alone did induce noticeable levels of caspase-3/-7 activity, cotreatment with β-thujaplicin significantly enhanced this activity. It seems that the β-thujaplicin-mediated decrease in XIAP protein levels contributes to the enhancement of caspase-3/-7 activity in TRAIL-treated NCI-H460 cells. The mechanism underlying the β-thujaplicin-induced decrease in XIAP levels remains to be elucidated. Interestingly, Makhov et al. [18] analyzed the action of TPEN on XIAP protein and their results implied that the addition of zinc chelators triggers a serine protease-dependent reduction of XIAP. It should be noted, however, that further experiments will be necessary to determine how β-thujaplicin decreases XIAP protein levels.

The BIR3 domain of XIAP, i.e., where caspase-9 and Smac proteins bind, is a promising site for the design of small-molecule XIAP inhibitors. Embelin, a small-molecule inhibitor that targets the XIAP-BIR3 domain [19], has a similar structure to β-thujaplicin, i.e., an aromatic ring with an α-hydroxy ketone and an alkyl group. Therefore, using a fluorescence polarization assay, we evaluated the inhibitory effect of tropolone derivatives on the binding of the Smac N-terminal peptide to the XIAP-BIR3 domain; both β- and γ-thujaplicins inhibited this binding in a dose-dependent manner and their inhibitory activities were greater than that of tropolone. These results suggest that the attachment of an isopropyl group to tropolone contributes to the binding activity to the XIAP-BIR3 domain.

Some synthetic β-thujaplicin derivatives have been reported to have higher cytotoxic effects than β-thujaplicin itself in cancer cell lines including NCI-H460 cells [3]. Thus, more potent XIAP-depleting and XIAP-BIR3-binding tropolone derivatives may exist. A trial was recently conducted in which dual action compounds, consisting of a Smac mimetic moiety and zinc-chelating moiety, were constructed [24]. The results of the present study reveal that β-thujaplicin might enhance TRAIL-induced apoptosis in NCI-H460 cells through the dual effects on XIAP, i.e., through XIAP depletion and binding to the XIAP-BIR3 domain. Thus, a tropolone scaffold may be useful for designing novel nonpeptidic small-molecular XIAP inhibitors that exert dual effects on XIAP in a single structure. Indeed, the screening and design of novel XIAP-inhibiting tropolone derivatives is currently in progress with tropolone being used as a starting compound. In future studies, the mechanism by which XIAP levels are reduced by tropolone derivatives should be investigated using more potent XIAP inhibitors derived from the tropolone structure.

## Figures and Tables

**Table 1:** Effects of tropolone derivatives on the binding of the Smac N-terminal peptide to the XIAP-BIR3 domain.

Compound	Structure	IC_50_ (μM) ^a^
Tropolone	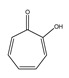	83.1 ± 6.9
β-thujaplicin	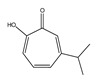	58.5 ± 2.8
γ-thujaplicin	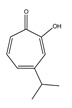	56.2 ± 2.9
Embelin	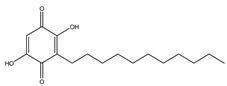	3.1 ± 0.6

^a^ The IC_50_, i.e., the inhibitor concentration at which 50% of bound Smac/Diablo peptide probe is displaced, was determined from the dose–response curve. Data represent the mean ± standard deviation of three independent experiments.
